# The Renaissance of Plant Mucilage in Health Promotion and Industrial Applications: A Review

**DOI:** 10.3390/nu13103354

**Published:** 2021-09-24

**Authors:** Katarzyna Dybka-Stępień, Anna Otlewska, Patrycja Góźdź, Małgorzata Piotrowska

**Affiliations:** Institute of Fermentation Technology and Microbiology, Faculty of Biotechnology and Food Sciences, Lodz University of Technology, Wólczańska 171/173, 90-530 Lodz, Poland; patrycjagozdz1@gmail.com (P.G.); malgorzata.piotrowska@p.lodz.pl (M.P.)

**Keywords:** plant mucilage, hydrocolloids, dietary fiber, nutraceuticals, health-promoting properties, food additives, prebiotics

## Abstract

Plant mucilage is a renewable and cost-effective source of plant-based compounds that are biologically active, biodegradable, biocompatible, nontoxic, and environmentally friendly. Until recently, plant mucilage has been of interest mostly for technological purposes. This review examined both its traditional uses and potential modern applications in a new generation of health-promoting foods, as well as in cosmetics and biomaterials. We explored the nutritional, phytochemical, and pharmacological richness of plant mucilage, with a particular focus on its biological activity. We also highlighted areas where more research is needed in order to understand the full commercial potential of plant mucilage.

## 1. Introduction

**Figure 1:**
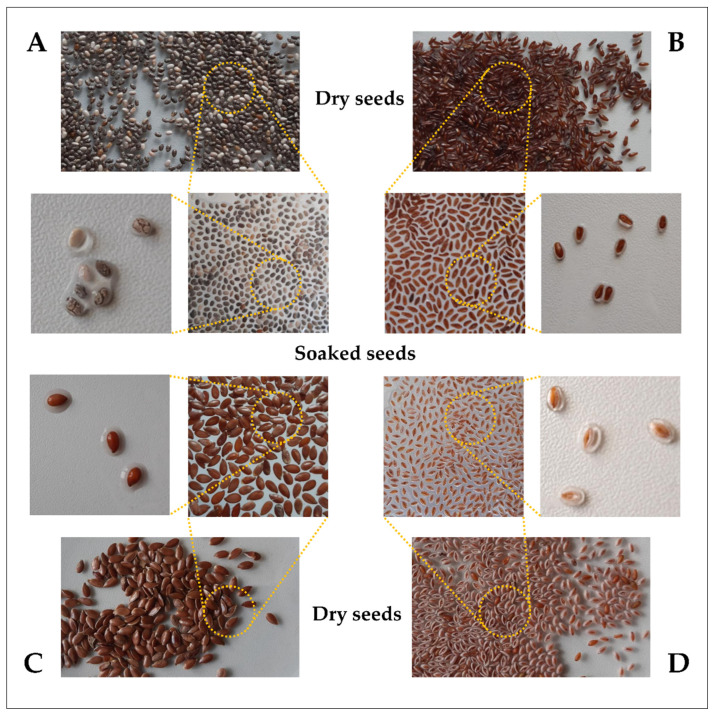
Seeds before and after swelling in water: (**A**) *Salvia hispanica*, (**B**) *Plantago afra*, (**C**) *Linum usitatissimum*, (**D**) *Plantago ovata*.

Plants have always played an important role in human health and nutrition. In recent years, there has been increasing interest in plant-derived compounds that are biologically active, biodegradable, biocompatible, nontoxic, and environmentally friendly. There has also been greater emphasis on finding renewable and cost-effective sources of plant-based products. These criteria are met by plant mucilage—a polysaccharide hydrocolloid with unique properties. Mucilage can occur directly as a jelly-like structure in the vegetative parts of plants (fruit, leaf, flower, root, or stem) as well as in seeds after treatment with water (Figure 1).

From a chemical perspective, mucilages are large molecules, containing mainly carbohydrates and uronic acids, as well as glycoproteins and other bioactive compounds [1,2]. Mucilages have a wide range of applications: in food and nutraceuticals as structuring, gelling, texturing, and film-forming agents, in pharmaceuticals as binders and disintegrants for drug delivery systems, and in cosmetics as stabilizers. They have also attracted great interest in the textile and paper industries, and can be used in the production of paint.

There are many well-known mucilages of plant origin, but new sources of these valuable hydrocolloids are still being sought. Table 1 presents 33 plant species belonging to 11 families, described in the literature, as rich sources of mucilage. The characteristics of mucilages depend both on their source (the seeds, leaves, roots, flowers, fruits, etc.) and the method of extraction [1,3].

**Figure 2:**
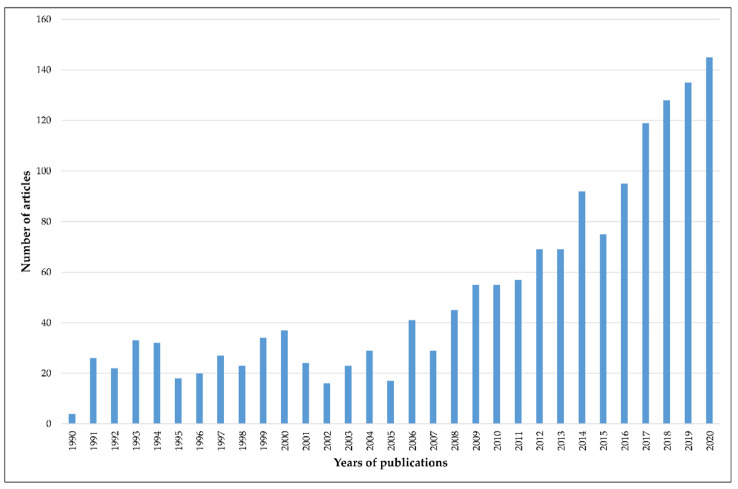
The number of publications in the last three decades regarding plant-derived mucilage based on Web of Science Core Collection.

Before 1991, there was little commercial and scientific interest in plant-derived mucilage and few publications on the topic (on average, three publications a year). The first scientific publications focused on identifying sources of plant mucilage and provided (often only qualitative) analyses of its chemical composition. In 1991–2000, interest in plant mucilages increased, with between 18 and 37 research articles per year. Although interest dipped again, since 2008 interest in the subject of plant-derived mucilages has been growing continuously (Figure 2). The development of analytical tools and greater understanding of the chemical structure and properties of mucilage has opened the way for innovative applications of this versatile material.

Although many papers have been published in the last twenty years, numerous authors have presented only the basic information about mucilage’s chemical composition and properties. In this review, we discussed the chemical composition as well as nutritional value of plant mucilages, as well as their new and potential applications. For this purpose, the first section is focused on holistic overview of plant-derived mucilages. The second section provides detailed information about 14 plant source of mucilages, their properties, and versatile applications, particularly in the food industry and in health promotion.

## 2. Materials and Methods

A preliminary review of the literature, based on keywords (plant mucilage, hydrocolloids, dietary fiber, nutraceuticals, health-promoting properties, food additives, prebiotics) in combination with the botanical names of plants known as sources of mucilage, identified about 1200 articles, book chapters, and conference papers in scientific databases (Scopus, Science Direct, Web of Science). The primary investigation was conducted based on the title, keywords, and abstract. In the next step, duplicates, articles published in languages other than English, and articles for which the full-texts are unavailable were excluded. Additionally, the conference papers without the peer-review process were omitted. In total, 234 scientific papers and book chapters published between 1990 and 2021 were selected for discussion in this literature review. The selected publications allowed us to answer the following questions: (1) what are the trends in this research area; (2) what is the level of interest in terms of the number of publications; (3) what are the areas of traditional application of plant-derived mucilages; and (4) what are the innovative directions of mucilage usage in food production and health promotion?

## 3. General Aspects of Plant-Derived Mucilages

### 3.1. Chemical Characterization of Mucilages

**Figure 3:**
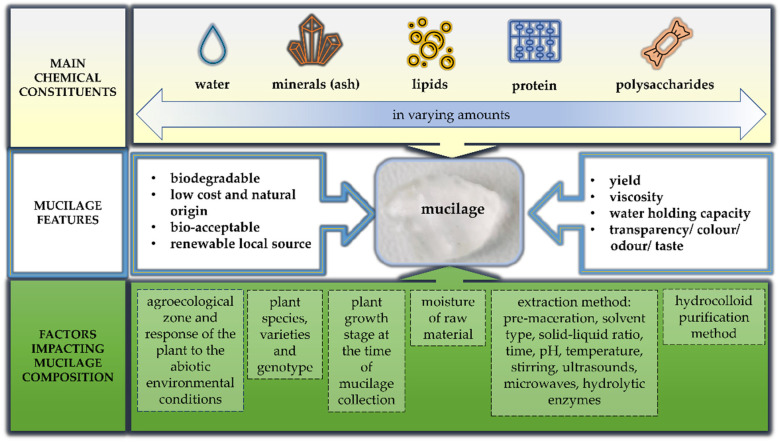
Parameters affecting the nutritional value and technological features of plant-derived mucilage.

The properties of plant-derived mucilages depend on their chemical structures and compositions. In recent years, extensive efforts have been made to link the quality (nutritional value, functional and technological features) of plant mucilages to their chemical compositions [2,53,54,55]. The important attributes of mucilages and factors impacting their quality are summarized in Figure 3. Mucilage is a complex polymeric substance composed mainly of heterogeneous carbohydrates in a highly branched structure. The most common are neutral and acidic polysaccharides, whose main backbone is composed of monomers such as D-xylose, L-arabinose, D-galactose, L-rhamnose, L-mannose, D-glucose, or L-fucose linked by glycosidic bonds. Uronic acids (D-galacturonic acid or D-glucuronic acid) can also be included. The plant species and extraction method influence the ratio of abovementioned individual constituents. Depending on the type of monomers and their proportions, glucomannan, galactomannan, arabinogalactan, rhamnogalacturonan, arabinoxylan, glucuronoxylan are distinguished [56,57].

Apart from polysaccharides, the basic components of plant mucilage are different proteins, lipids, minerals, and water. Minor components found in mucilage include tannins, flavonoids, sterols, and alkaloids. The concentration of protein in mucilage has an impact on its water-holding capacity. Higher protein concentrations in mucilage are known to improve the texture and consistency of products containing mucilage. The chemical composition of hydrocolloids may promote the growth of unwanted microorganisms and increase the instability of mucilage. More research is needed on the relation between plant species and varieties, and the polysaccharide composition and yield of mucilage. Other important factors influencing the chemical composition of mucilage are agronomical variations associated with local climatic conditions (temperature, humidity, insolation) and soil and hydration parameters (such as the availability of nutrients, pH, salinity, draught etc.). In some regions, possible heavy metal contamination from the soil should also be considered. The morphological and physiological features of the plant parts can significantly affect the nutritional value and physiochemical parameters of the harvested hydrocolloid. The extraction and purification methods used are also important parameters. Many studies use conventional aqueous (cold/hot) or alkali extraction, supported with acid pre-treatment as well as enzymatic, ultrasound, and microwave treatment of the raw material. “Green” techniques used for the extraction of plant biological compounds have recently received more attention, improving extraction yield, reducing process time and the need for chemicals, and lowing costs. Further research in needed on optimizing the parameters of mucilage extraction from plants [2,53,54,55].

### 3.2. Function of Mucilages in Plants

Mucilages have many important biological functions in plant depending on their chemical composition and location in plant organs. Mucilages are essential for adaptation to diverse environments and can be secreted by cells known as mucilage-secreting cells (MSCs) in seeds, fruits, flowers, leaves, stems, bark, and roots [58,59,60]. The production of seed (myxospermy) and fruit (myxocarpy) mucilages ensures adequate hydration of these plant organs, which is especially important in desert environments. As well as maintaining hydration, mucilages play a role in maintaining water and oxygen in seed tissues. Due to the presence of polysaccharides in their chemical composition, they are a source of energy [2,61]. Mucilages are thus important for seed germination. In a study by Huang et al., it was observed that hydration and mucilage production had a positive effect on germination of *Lepidium perfoliatum* seeds. Mucilage-coated seeds sprouted faster, within 1–3 days, and were larger than smaller seeds without mucilage, which germinated about 3 days later [62]. The adhesive properties of mucilages also play a crucial role, increasing the surface area and facilitating the attachment of the diaspores to the soil. Adhesion to animal fur or feathers enables plant dispersal and spread. At the same time, mucilage protects against digestion by animals, insects, and pathogens [63]. Mucilage in leaves and stems functions mainly as a store of water and nutrients [64]. Mucilage also attracts insects, which can be a source of nitrogen, and protects against herbivores [58]. In cactus stems, mucilages may protect against low temperatures [65]. In flowering plants of the *Orchidaceae*, mucilage maintains hydration [66]. According to a study by Cassola et al. on *Elleanthus brasiliensis*, mucilage can protect against pathogens and herbivores due to the presence of terpenes and other compounds secreted by the inflorescences [67]. In addition to taking up and storing water and nutrients, including macro- and micronutrients, the mucilages produced by plant roots protect against toxic compounds. Secreted mainly by border cells and root cap cells, they protect root during growth in the soil by maintaining moisture [2,68]. In the case of climbing plants such as *Hedera helix*, secretion of adhesive mucilages by adventitious roots enables them to overgrow different surfaces, e.g., trees, rocks, and fences [58]. Due to the presence of carbon sources in their composition, mucilages can provide the proper conditions for the growth of root microbiota. The mucilages produced by pea roots have been shown to be suitable for rhizosphere bacteria such as *Rhizobium* sp. and *Pseudomonas* sp. [68].

### 3.3. Application of Plant-Derived Mucilage

Understanding the chemical structure and physical characteristics of mucilage is crucial to determining its potential functional and health-promoting properties [1,3]. As already mentioned, the major compounds in plant-derived mucilages are polysaccharides. Therefore, the molecular weight of polysaccharides and their distribution determine the technological (rheological and emulsification) properties of mucilages. Mucilages containing polysaccharides with high molecular weight can be used as thickening and structuring (gel-forming) agents in food, especially gluten-free products (bread, pasta), as they increase the flexibility and viscosity of continuous phase [1,69,70]. Moreover, mucilage (mainly from chia seeds) is used as a fat replacer in bakery products (e.g., bread and cake) and dairy products (e.g., yogurt, cheese, and ice cream) [71,72]. Many studies have shown the stabilizing properties of mucilages (chia or basil seeds) in various foods, including ice cream and salad dressing. Edible films and coatings based on plant mucilage offer a promising alternative to chemical substances applied to extend the shelf-life of fruits, fish, and meat during storage [73].

Due to the fact that mucilages are biocompatible and nontoxic, they can be successfully applied in the pharmaceutical industry, primarily in drug-delivery systems. They constitute a binder or disintegrant for the targeted and sustained release of drugs during tablet formulation (e.g., *Aloe vera*, *Cassia tora*, *Moringa oleifera*) [57,74]. Furthermore, mucilages can be used as thickeners and protective colloids in liquids and suspensions and as gel-forming agents in gels [74]. The thickening abilities of mucilage also make them useful in cosmetics. Mucilages (from quince and aloe) are used as moisturizers in creams, lotions, and soaps. Their water-holding ability together with moisturizing and antimicrobial properties make mucilages suitable for the production of dressings for wounds and skin burns, as well as to treat inflammation [1,75].

The bioactive and nutraceutical properties, as well as their potential health benefits, make plant mucilages valuable ingredients in healthy food. Mucilages are primarily a rich source of dietary fiber. Thus, their intake can have a laxative effect, regulate satiety, counteract hyperlipidaemia and hyperglycaemia, and reduce obesity. Seed mucilages have been reported to modulate intestinal microbiota due to their prebiotic activity. The antioxidant and immunomodulatory effects of mucilages have also been proven [3,53]. Seed mucilages may reduce the risk of colon cancer and rectal cancer, and could help protect against coronary heart diseases [76].

## 4. Selected Plants as a Mucilage Origin in the Light of Recent Research

### 4.1. Salvia

Salvia (sage), from the family *Lamiaceae*, comprises about 1000 species and is well-recognized for its medical, food, and feed applications [77]. Two species of sage—*Salvia hispanica* and *S. macrosiphon*—have attracted the greatest interest from researchers and industry.

*Salvia hispanica* (chia) is an annual herbaceous plant, which originates in Mexico and Central America [78,79]. Chia seeds (also known as chia nutlets) are oval-shaped and white, beige-white to dark brown, or even black in color. The typical dimensions of the nutlets are 1.87–2.15 mm length, 1.21–1.40 mm width, and 0.80–0.88 mm thickness [80,81]. When immersed in water, chia seeds release polysaccharide mucilage. Various studies have been carried out regarding the nutritional and medicinal values as well as technological and sensory properties of chia seeds and their derivatives, including chia mucilage (CM). Chia seeds are rich in omega-3 fatty acids and other polyunsaturated fatty acids. They also contain fiber, protein, and antioxidants such as quercetin, myricetin, caffeic acid, chlorogenic acid, rosmarinic acid, and other biologically active and health-promoting compounds [72,82,83]. Chia mucilage is composed of carbohydrates (including xylose, mannose, glucose, galactose and arabinose) and uronic acids (glucuronic and galacturonic acids), protein, fat, and ash. In general, CM is considered a tetrasaccharide consisting of β-D-xylopyranosyl, α-D-glucopyranosyl, and 4-*O*-methyl-α-D-glucopyranosyluronic acid. However, some physicochemical variation is observed depending on the mucilage extraction method used as well as on the seed variety and geographical source [84]. Chia mucilage contains planteose, a health-promoting galactosyl-sucrose oligosaccharide (GSO). First identified in psyllium, GSOs are prebiotics that stimulate the growth of beneficial bacteria in the gastrointestinal tract [85].

Chia mucilage serves as a multifunctional food additive, which can be used to replace fat, eggs, and gluten [84]. Recently, chia mucilage has been employed in the production of low-fat foods such as yogurt [72], bread and bakery products, and meat products [86,87]. Consumers accepted the 7.5% addition of CM to yogurts, and rheological measurements confirmed that the fortified yogurts had better consistency, firmness, viscosity, and stress resistance compared with full-fat and skimmed yogurts [72]. Atik et al. studied the effects of the application of chia mucilage on the technological, sensory, and microstructural properties of yogurts supported with CM or guar gum [88]. Aside from being a good texture stabilizer, improving the firmness and consistency of the yogurt, CM showed better antioxidant properties than guar gum. The potential of CM as a stabilizer in ice cream production was proven by the addition of 0.1–0.35 (*w/w*) freeze-dried CM replacing commercial stabilizer in ice cream [88]. These studies open the way for designing novel skimmed yogurts and desserts with higher fiber content based on CM.

The ability of CM to form protein-stabilized emulsion gel makes it an attractive replacement for animal fat, e.g., in reduced-fat frankfurters [89]. The technological and functional properties of CM added to Bologna sausages have been discussed by Câmara et al. [87]. Chia mucilage powder and chia mucilage gel were used in sausage recipes with reduced fat and phosphate contents. It was shown that the addition of 2% CM gel allowed for the replacement of 50% of the fat and the total removal of phosphate in the final product. Reduced-fat longanizas containing CM were formulated by Pintado et al. [90]. Chia mucilage has also been proposed as a vegan mayonnaise texture stabilizer [91].

Chia mucilage offers a promising replacement for egg in chocolate cake [92]. Fernandes et al. prepared a ready-made cake-mix with lower fat (60.4% reduction) and increased protein content [71]. Chia mucilage has also been proposed as a fat reducer in pound cakes [93]. Low-fat cookies containing CM were prepared by Rocha et al. [83]. Chia mucilage-stabilized muffins composed of chia seeds, water, inulin, hemp, and flaxseed oil were analyzed by Gutierrez-Luna et al. [83,94]. In another study, the technological quality of breads were improved when wheat flour was substituted with 5% and 10% (*w/w*) chia flour with mucilage [95]. It should be emphasized that the addition of chia flour and mucilage also improved the water absorption, dough development time, and the stability of the mixes during the preparation of bread. Chia mucilage has been investigated as an agent to decrease the glycemic index of bakery products and as a texturizing agent in gluten-free bread [96,97]. Rice flour fortified with CM has been used in gluten-free pasta [98].

There is increasing interest in the use of CM for encapsulation. Recently, Renteria-Ortega et al. applied electrohydrodynamic atomization (EHDA; electrospraying) to obtain CM-sodium alginate particles with different morphological and mechanical characteristics, depending on the formulation [99,100]. In another study, de Campo et al. used CM to synthetize stable chia seed oil nanoparticles (CSO-NP) [101]. Mucilage and protein extracted from chia were shown to be useful encapsulating agents for probiotics, ensuring high viability, whereas an antimicrobial effect was achieved by encapsulating green cardamomum essential oils with chia mucilage polyvinyl alcohol (CM/PVA) blends [102,103].

Chia mucilage was found to be suitable for the production of edible and biodegradable films [104]. Thin films made of CM and whey protein concentrate were produced and characterized by Munoz et al. [105]. The CM-protein fraction of the seed, supplemented with clove essential oil, was used to prepare biodegradable packaging film [106]. Edible chia mucilage/gelatin (CM/G) films with antimicrobial properties, containing oregano essential oils, have potential uses in the food and pharmaceutical industries [107]. Blended CM and nanocellulose fibers (added at 3% and 6% concentrations) were categorized as biodegradable, biocompatible, nontoxic, antioxidative and antimicrobial [108]. In powdered form, CM has been reported to be super-disintegrant, making it suitable for use in fast-dissolving tablets [109].

Mucilage from chia nutlets can be extracted in different ways, including by hot/cold extraction at various temperatures, using different solvents, agitation and seed-solvent contact times, or by oven-drying and freeze-drying with different parameters. These parameters strongly affect the extraction yields and properties of CM [110]. The optimization of CM extraction yield was described by Chiang et al. [84]. Chia mucilage is typically extracted using methods including at least one hot extraction stage. Yields vary from 5% to 16% [78]. Tavares and colleagues identified the optimal cold extraction seed to water ratio as 1:20, resulting in 8.46% mucilage yield at room temperature (27 °C) [78]. Ultrasonic-assisted removal of the CM mucilage envelope prior to the extraction of the oil by pressurized liquid extraction (PLE) has also been described [111].

Chia mucilage suspensions are susceptible to degradation during storage, affecting their rheological properties. Low concentrations of ascorbic acid and lemongrass essential oil can have a protective effect on chia hydrocolloidal mucilage, as demonstrated by Cuomo et al. [112,113].

*Salvia macrosiphon* (wild sage) is used as a fiber source as well as for medicinal purposes due to its chemical composition, biological activity, and multifunctional applications. Wild sage seed mucilage (WSSM), also known as sage seed gum (SSG) or macrosiphon seed mucilage (MSM), has great value for use in a food and packaging products. In recent years, WSSM-based pasta and apple cake have been developed with lower glycemic indexes [114]. Moreover, WSSM blended with sodium alginate has been shown to be good packaging material for *Lacticaseibacillus casei* encapsulation. The chemical composition of this mucilage was found to provide a prebiotic effect linked with higher resistance of probiotic bacteria to harsh gastrointestinal conditions [115]. Wild sage seed mucilage-based food coatings have been investigated by several researchers. Edible mucilage films were prepared by Davachi et al. with 2% nanoclay to improve their mechanical, physical, and thermal features [116]. The films were antibacterial, making them a promising bio-degradable packaging material. When WSSM was combined with chitosan, improved antimicrobial, mechanical, and barrier properties were achieved [117]. According to Bostan et al., powdered WSSM contains 80% carbohydrates, 2.8% proteins, 0.9% lipids, 6.7% moisture, 1.7% crude fiber, and 8.2% ash [118]. The method used for mucilage extraction was found to significantly impact the yield and chemical composition.

### 4.2. Ocimum basilium

*Ocimum basilium* (basil) is a culinary herb of the family *Lamiaceae*, which is used as a spice in cuisines worldwide. Basil is native to the tropical regions of Asia, Africa, and Central and South America, but is now a commercially cultivated plant. Basil leaves contain bioactive compounds such as flavonoids and have antioxidant and antimicrobial activities [119]. In aqueous solution, basil seeds produce mucilage—basil seed mucilage (BSM) or basil seed gum (BSG)—which can be a natural source of polysaccharides and soluble fiber. According to Nazir et al., seed extraction with water at a temperature of 60 °C over 1.6 h provides a high yield of BSM, of about 20.5 g/100 g seed dry mass. The total dietary fiber content observed for basil seed mucilage was 98.5% [6,95].

The polysaccharide extracted from basil seed contains high-molecular-weight polysaccharides (2320 kDa), which are composed of acidic and neutral polysaccharides in a ratio of 1:1. Basil seed gum consists of glucose, galacturonic acid, rhamnose, mannose, arabinose, glucuronic acid, and galacturonic acidic polysaccharides. The neutral polysaccharide fraction shows a high abundance of terminal β-linked D-galactopyranose moieties [120].

Basil seed mucilage has many desirable properties, such as hydrophilicity, biocompatibility, low production cost, film forming capability, edibility, and viscoelastic properties [121]. These properties make it suitable for use as a food stabilizer, emulsifier, and texture improver. Basil seed mucilage can be used as an edible film or coating for various foods, a stabilizer in mayonnaise, or as a substitute for fat in low-fat ice creams and cakes, improving the quality and health properties of these food products [91,100,122]. Crude basil oligosaccharide extract prepared by enzymatic hydrolysis possesses prebiotic properties. Growth of lactic acid bacteria (LAB) has been stimulated by basil oligosaccharide extracts, in both in vitro and in vivo studies, while *Salmonella* growth was decreased [123]. Ghasempour et al. demonstrated that the addition of 0.4% BSG to probiotic yogurt improved its antioxidant activity, textural properties, and lactic acid bacteria viability [124].

Mucilage from *Ocimum basilicum* seeds exhibits antitumor and antidiabetic activity. Polysaccharides obtained by water extraction followed by ethanol precipitation inhibited the invasiveness and progression of a malignant hepatocellular carcinoma tumor [125,126]. Gajendiran et al. found that the petroleum ether and methanol extracts from BSM had antibacterial effects against pathogenic bacteria, such as *Pseudomonas aeruginosa*, *Escherichia coli*, *Shigella dysenteriae*, and *Klebsiella pneumoniae* [127].

### 4.3. Hyptis suaveolens

*Hyptis suaveolens*, syn. *Mesosphaerum suaveolens* (common names bushmint, pignut, or chan) is an aromatic herb belonging to the *Lamiaceae* family. It occurs naturally in the tropical and subtropical regions of Latin America, India, China, Australia and Africa where is used in food; it is, however, considered as a worldwide weed [7].

*Hyptis suaveolens* seeds soaked in water produce mucilage (HSM), which contains neutral and acidic polysaccharide fraction in the ratio of 1:1 [128]. According to Morales-Tovar et al., the optimal conditions for water mucilage extraction are under mechanical agitation for 14 min at 50 °C, with a seeds to water ratio of 1:40. Extraction with ultrasound at 34 °C for 30 min resulted in increased extraction yield and improved HSM parameters such as viscosity and heat capacity [89]. The neutral polysaccharide consists of the monosaccharides galactose, glucose, and mannose. The acidic fraction is composed of fucose, xylose, and 4-*O*-methylglucuronic acid units [129]. The neutral HSM fraction is composed of D-galactopyranosyl, D-glucopyranosyl, and D-mannopyranosyl units. The acid polysaccharide contains L-fucopyranosyl, D-xylopyranosyl, and 4-*O*-methyl-D-glucuronic acid units [130].

Due to its high water-binding capacity, swelling, viscosity, and unique chemical composition, HSM has found wide applications as an emulsifier and gelling agent, stabilizer, binder, or disintegrant in food, pharmaceuticals, and other products [129,131]. The mucilage of *Hyptis suaveolens* could also have prebiotic abilities. Mueller et al. found that the neutral polysaccharide fraction enhances the growth of lactic acid bacteria, mainly probiotic lactobacilli, e.g., *Lacticaseibacillus paracasei*, *Lacticaseibacillus rhamnosus*, *Lactiplantibacillus plantarum*, *Levilactobacillus brevis*, and *Limosilactobacillus fermentum*. The externally located galactose units of the side chains, which are more available for the β-galactosidase enzyme, are responsible for this effect. In contrast, the acidic fraction showed no prebiotic activity, which may be due to the reduced availability for fermentation by probiotic strains of the branched xylose units [8].

### 4.4. Plantago

*Plantago* is one of the genera belonging to the family *Plantaginaceae*, which are common herbs used widely as medicinal and nutritional agents [132]. Of the many species (more than 200), the most widely used for industrial and medical purposes are *Plantago arenaria* (*P. indica*), *P. asiatica*, *P. major*, *P. lanceolata* (narrow-leaf plantain), *P. notata*, *P. ovata* (blond psyllium, isabgul, ispaghula), and *P. psyllium* (also called psyllium) [133,134]. Due to its remarkable features, *Plantago* mucilage is used around the world to treat a wide spectrum of health disorders. It has been proven that polysaccharides derived from these plants reduce cholesterol and blood glucose, have anti-inflammatory and antioxidant activities, and are anticarcinogenic. They are also a good source of dietary fiber [135,136]. Based on the antiallergic and antimicrobial properties of mucilage from *P. ovata*, a mouthwash containing polysaccharides and vinegar was developed. Successful preliminary studies were conducted treating oral mucositis in patients with breast cancer undergoing chemotherapy [137]. Mucilage can be assist wound healing. Polysaccharides derived from *P. ovata* were found to be an excellent superdisintegrant and suspending agent for drug-delivery systems [138]. *Plantago* can be applied in different forms—seeds, husks (also known as isabgol) with the epidermidis (dried seed coat) removed, leaves, oil, or mucilage. The seeds contain between 10% and 30% of mucilage (also commonly referred to as gum in the literature), which forms a specific multilayered coating surrounding the seed [139]. The hydrocolloids in the plants may differ significantly in terms of the rheological properties of the mucilage (from the weak gel formed by *P. asiatica* [140] to the strong gelling behavior observed for *P. ovata* [141]), its three-dimensional structure, and its biological activity depending on the *Plantago* species, the chemical composition of the mucilage, and the extraction method used (mainly solvents such as hot water, cold water, or alkali).

Arabinoxylan is a typical polysaccharide found in *Plantago*. Many reports describe its unusual branched structure β-(1,4) or β-(1,4)/(1,3)-linked xylopyranose backbones and the atypical linkage composition characteristic for *Plantago* seed mucilage. Cowley et al. provided a detailed analysis of the morphometric parameters and chemical constituents of *Planatgo* seed, the extraction yield, and the chemical composition and structure of fractionated mucilage derived from 12 *Plantago* species [142]. *Plantago* mucilage is a source of protein, dietary fiber, and saturated fatty acids. It is also a rich source of both ω-3 and ω-6 polyunsaturated acids (>78% of total fatty acids). The monosaccharides found in the hydrocolloid fractions include xylose, arabinose, rhamnose, galactose, glucose and small amounts of mannose, galactose, and galacturonic and glucuronic acids [142]. In the case of *P. lanceolata*, *P. ovata*, and *P. media*, additional cellulose fibrils form a regular, radially arranged skeleton of pectin, which surrounds the seed surface [143].

As well as being medical plants, *Plantago* have many nutritional benefits and technological properties that can be useful (for the production of bakery products, jams and jellies, vegan mayonnaise, dairy products, etc.) [144]. Recently, *P. ovata* seed mucilage was demonstrated to act not only as a fat replacer in yogurt but also as a prebiotic [145]. *Plantago* mucilage has been proposed as a biosorbent in low-cost technology to remove heavy metal from water. It is also a tissue-protective chelating agent when taken orally [146,147]. Like mucilages from flaxseed, plantain, basil, and chia, *Plantago* mucilage can be used as an environmentally friendly nontoxic natural glue [141].

### 4.5. Trigonella foenum-graecum

Fenugreek is also known as Greek hay, Greek clover, Greek buttercup, divine grass, or ox horn. Widely cultivated since ancient times, the plant was imported from Greece by the Romans [148,149]. Fenugreek is an annual herbaceous plant belonging to the *Fabaceae* family. There are 97 species, of which the most common worldwide is *Trigonella foenum-graecum* [148,150]. Fenugreek is grown almost all over the world, mainly in Europe, Asia, Africa, and Australia, where it is used as a spice [148]. Its seeds and leaves are characterized by the intense aroma and flavor [151]. The brownish-gold seeds of fenugreek are mainly tetrahedral or oval in shape, measuring 3–5 mm in length and 2–3 mm in width [148]. Due to its rich composition and properties, fenugreek is of great interest for use in functional foods. Fenugreek seeds contain steroidal saponins, aglycones (diosgenin, jamogenin, tigogenin, neotigogenin, gitogenin, neogitogenin), and flavonoids (vitexin, isovitexin, vicenin, saponarin, luteolin, tricin, quercetin, naringenin, kaempferol) [149]. They are also rich in minerals (potassium, magnesium, calcium), carbohydrates, amino acids (aspartic and glutamic acid), and fatty acids (linoleic acid, α-linolenic acid) [152]. The mucilage of fenugreek seeds consists mainly of galactomannans.

Isolation of mucilages mostly involves a boiling step, followed by the use of solvents. In a study by Iurian et al., mucilages were extracted using acetone. The high-performance liquid chromatography (HPLC) analysis showed the presence of galactose (41.7%), mannose (34.7%), and non-hydrolyzing material (23.6%) [153]. In a study by Verma et al., isolations were conducted using ethanol. Carbohydrates were observed and the pH of the mucilage was 7.9 [154]. Many studies have been carried out on the potential applications of fenugreek mucilage in food, cosmetics and pharmaceuticals, etc. Studies have shown the mucilage to have high viscosity and very good emulsifying properties, probably due to the presence of low concentrations of proteins, making the mucilage suitable for use as a thickener and stabilizer [155,156]. In a study by Memiş et al., biodegradable films were made from fenugreek seed mucilage and nanoclay. The films with 5% nanoclay had high thermal and tensile strength, provided an oxygen barrier, and exhibited antimicrobial properties. Growth inhibitory effects were observed against *Staphylococcus aureus*, *Listeria monocytogenes*, *Bacillus cereus* and *Escherichia coli* O157:H7 [157]. The antioxidant and antimicrobial properties of fenugreek polysaccharides were studied by Wu et al. Antifungal activity was observed against *Botrytis cinerea*, *Fusarium moniliforme*, *Ascochyta fabae*, eggplant *Verticillicum wilt*. Inactivating properties were observed against hydroxyl radicals and superoxide anions were observed [158].

Fenugreek mucilage has been shown to have positive effects for the treatment of arthritis. A dose of 75 mg/kg of mucilage was better at reducing inflammatory swelling in rats than a commercial drug. Fenugreek mucilage inhibited the enzymes responsible for the development of inflammation (i.e., cyclooxygenase) [151]. Mucilage can be used as a drug carrier in nasal formulations, due to its mucoadhesive properties in low concentrations [159]. A study by Iurian et al. demonstrated the feasibility of using fenugreek mucilage as matrix component for pharmaceutical lyophilizates [153]. Tablets made from mucilage showed higher crushing strength and longer disintegration time compared with tablets made from gelatin. Tablets with 1% fenugreek mucilage showed an adequate disintegration to strength ratio [153]. Fenugreek seed gel has shown promising results in studies on encapsulation of probiotic bacteria. Microencapsulation of *Lactiplantibacillus plantarum* 15HN in a gel formulation (1.5% alginate with 0.5% fenugreek) provided adequate bacterial cell viability under low pH and high bile salt concentrations [160]. In a study by Zemzmi et al. on the effect of mucilage on rabbits, fermentation by cecal bacteria was observed and the gel was resistant to digestive enzymes in vitro, indicating the potential prebiotic effect of fenugreek seed gel extract [161].

### 4.6. Cassia

*Cassia* is a morphologically variable genus of plant, belonging to the family *Leguminosae* including both annual and perennial herbs (*Cassia obtusifolia*, *Cassia tora*), shrubs (*Cassia auriculata*), and trees (*Cassia fistula*) [162]. Due to its yellow flowers, *Cassia* is often cultivated as an ornamental plant. Species of *Cassia* are found in India (*Cassia tora*, *Cassia obtusifolia*, *Cassia uniflora*), China (*Cassia obtusifolia*, *Cassia fistula*), Korea (*Cassia obtusifolia*), and Japan (*Cassia obtusifolia*) [14,19,163,164]. Cassia has small diamond-shaped seeds (2–4 mm long), which are yellow-brown to dark brown in color [19].

Despite the extreme diversity within the genus, most *Cassia* species are used in traditional medicine [164]. The dry and mature seeds of *Cassia obtusifolia* or *Cassia tora* (known as Juemingzi in Chinese or Ketsumeishi in Japan) are used to improve eyesight, reduce hypertension and hyperlipidemia, and as a laxative, tonic, and diuretic [164,165]. *Cassia obtusifolia* seeds contain 18.5–22.9% crude protein, 5.3–7.4% crude lipid, 6.8–9.4% crude fiber, 5.1–5.8% ash, and 57.0–60.0% carbohydrate [163]. According to Deore and Mahajan, sugars constitute only 8% of carbohydrates, while low-water soluble gums account for 7% [164]. After soaking in water, the swelling seeds are the main source of mucilages. Recent oral toxicity analysis showed seed mucilage from *Cassia uniflora*, which means that it can be used in the food and pharmaceutical industries as a gelling and stabilizing agent or tablet binder [19]. Studies carried out by Singh et al. showed the usefulness of *C. tora* seed mucilages in concentrations of 2.0–8.0% (*w/v*) for the preparations of tablets [166]. The physico-chemical and organoleptic parameters of *C. fistula* and *C. obtusifolia* seed mucilages make them suitable mucoadhesive agents for drug-delivery applications [164,167].

### 4.7. Basella alba

Another group of plants is used as a source of mucilage, mainly from the leaves, stems, or flowers of genera belonging to the order *Caryophyllales*.

*Basella alba* is a heat-tolerant, edible, perennial vine plant abundant in tropical regions of Asia, Africa, and South America. *Basella alba* is a member of the *Basellaceae* family, and it is commonly known as Malabar, as well as Indian or Ceylon spinach. Its leaves are used as a vegetable in Asian cuisine [35]. *Basella alba* contains contain health-promoting substances, such as basellasaponins, peptides, phenolic compounds, carotenoids, organic acids, water soluble polysaccharides, and vitamins. All aerial structures of the plant, especially the leaves, are sources of *Basella alba* mucilage (BAM) [35]. Pareek et al. first treated *Basella alba* leaves with petroleum ether to defatten them, and then performed water extraction and precipitation with acetone, obtaining mucilage with a yield of 14.8% *w*/*w* dried mass and total carbohydrate content of 84.05% [168]. *Basella alba* mucilage is characterized by a pH ranging from 5.3 to 5.4 and mainly consists of polysaccharides. It contains D-galactose as a major monosaccharide and a small amount of L-arabinose, as well as water and acid insoluble ash, sulphated ash (1.35%), chloride, and uronic acid [168,169].

Due to its properties, such as strong suspending ability and high viscosity, BAM is used as a thickening and gelling agent in the food and pharmaceutical industries and also as a binder for uncoated tablets [35,170].

According to Das et al., BAM can be successfully used to encapsulate hydrophobic antioxidants, as shown by the example of curcumin. The capsules increased the solubility of curcumin in water, and thus its hydrophilicity, biocompatibility, and antioxidant activity in aqueous medium. The capsules were characterized by pH and photostability [171].

### 4.8. Spinacia oleracea

*Spinacia oleracea* (spinach) is a common annual or biennial edible plant belonging to the *Amaranthaceae* family. It is native to central and southwestern Asia. It is now also cultivated in all temperate and subtropical regions of Europe, Asia, and North America [36]. Spinach is a plant rich in various health-promoting compounds, e.g., flavones, flavanols, methylenedioxyflavonol glucuronides, glucuronides, and carotenoids. Therefore, it has antiobesity, antimutagenic, antioxidant, anticancer, hypoglycemic, and anti-inflammatory properties [172]. There are no literature data on mucilage obtained from this plant. However, according to Pal et al., the mucilage from *S. oleracea* leaves obtained by water extraction and acetone precipitation has potential to be used as an innovative suspending agent and adjuvant in pharmaceutical formulations [36].

### 4.9. Talinum triangulare

*Talinum triangulare* (syn. *T. fructiosum*) from the *Talinaceae* family is commonly called Ceylon Spinach or Waterleaf. It is a tropical cosmopolitan leafy vegetable well known in Africa, America, and Asia. In most parts of Africa, the leaves are pressed with or without salt to remove the mucilage and eaten after cooking. The mucilage is most often thrown away or used as a food additive [38].

The most common method of obtaining mucilage from waterleaf in research is water extraction. In a study by Adetuyi et al., the leaves were homogenized with water, filtered, and heated at 70 °C for 5 min. The mucilage was precipitated and washed with ethanol followed by acetone. The yield of mucilage was about 2.1% *w/w*. Unlike other mucilaginous plants, which contain mainly polysaccharides, the mucilage of *Talinum triangulare* contains proteins (54.3%), fat (29.0%), and a small amount of carbohydrates (5.4%) and fiber (3.5%) [38]. The optimum conditions for mucilage extraction from *T. paniculatum*, another mucilage-producing species were established by Nor et al. After water extraction at a temperature of 90 °C and pH of 8, mucilage was obtained with a yield of 3.4% and crude protein content of about 30% [173].

Mucilage from *Talinum* contains phytate, saponins, vitamin C, and phenolic compounds, thanks to which it exhibits antioxidant activity. Due to its composition, especially its protein content, *Talinum* mucilage could help prevent malnutrition in regions deficient in animal protein. As a source of pro-health antioxidants, it can also be used as a therapeutic agent to prevent oxidative stress-related diseases [38].

### 4.10. Opuntia

Plants of the genus *Opuntia* (commonly known as prickly pear), which belongs to the family *Cactaceae*, are among the most recognized cacti due to their distinctive edible fruits (62% of *Opuntia* crops) [41,65]. There are 377 known endemic genera, 104 of which occur in Mexico, where they are most common and most widely used cacti [174]. *Opuntia ficus-indica* is the most commonly cultivated species [175]. Opuntia has been used in traditional medicine in Mexico. The fruit and the cladodes are eaten both fresh and processed [176]. Beyond Mexico, *Opuntia* it is also found in Morocco, Argentina, Brazil, the USA, Peru, Bolivia, Italy, Spain, Israel, Africa, Australia, and Canada. Opuntia easily adapts to the environment, so it is able to grow in desert and semi-desert areas and at temperatures of 5 °C and down to −40 °C (Canada). Survival in adverse conditions is ensured by the production of mucilage [41].

The mucilages present in the fruit (OFM), cladodes (OCM), and in the peel differ in terms of their chemical composition. The weight of the prickly pear fruit is in the range of 100–150 g, and the elongated cladodes 40–100 g with widths of about 20 cm and lengths of 30–80 cm. Mucilages isolated from the pulp and pear are mainly composed of carbohydrates (64.15% and 93.48%), which are responsible for the functions and physicochemical properties of *Opuntia*. The cladodes contain rhamnose, arabinose, galactose, galacturonic acid, xylose, galactose, and glucose. The mucilage of the fruit and peel consists mainly of rhamnose and galacturonic acid. The protein content in OFM is 0.86%, while OCM is 1.04%. The protein content affects emulsification and stabilization. Mucilages are also rich in minerals (Mg, Fe, Ca, Zn, Mn, K, Na), and so they can have positive effects on emulsification, viscosity, and enzymatic activity. Fatty acids such as linoleic and α-linolenic acids are also present in the mucilage powders [177]. Uronic acid (23.4%) is present in OFM [41].

Extraction methods using water and heating have been reported in the literature. Boiling and centrifugation have been used to isolate the mucilage from *Opuntia* organs [177,178]. Ethanol has been investigated as a potential solvent in the drying stage [174,179]. The compositions of the mucilages extracted from the fruit by methods using water and chemical solvents are reported as being similar. Similar results have also been observed between the yields of the aqueous extract obtained from wet fruit pulp (0.48%) and by acid/alcohol extraction (0.46%). This demonstrates the possibility of using environmentally friendly and healthy methods of mucilage extraction [41]. A study by du Toit et al. analyzed the composition of mucilage powders according to harvest time. It was observed that mucilage from plant harvested in February contained the highest amounts of minerals and lowest amounts of carbohydrates. The highest amounts of carbohydrates were obtained in June. This indicates the influence of environmental conditions on mucilage production and production according to dietary requirements [177]. *Opuntia* mucilage has potential for use in the food and pharmaceutical industries. According to Liguori et al., *Opuntia ficus-indica* mucilage can be used as an alternative to water in bread production. The mucilage shows no inhibitory effect on either baker’s yeast or lactic fermentation bacteria, which is important during the fermentation stage. Polyphenols contained in prickly pear extract show antioxidant and antimicrobial properties against *Salmonella* spp., *Staphylococcus aureus*, *Bacillus cereus*, *Pseudomonas aeruginosa*, and *Escherichia coli*. Bread made with added prickly pear mucilage had a higher phenolic concentration than bread baked without the mucilage, and no negative effect on the technological properties of bread was observed. These results suggest that *Opuntia* the mucilage can be applied in the food industry [178]. The potential prebiotic effect of *Opuntia* gum powder was proven in a study by Cruz-Rubio et al. The mucilage heteropolysaccharides were also shown to be a fermentable carbon source. The best prebiotic effects were obtained for powder dried for 96 h at 50 °C. The study was conducted on the bacterial strains *Bifidobacterium longum* subsp. *infantis*, *Lacticaseibacillus rhamnosus*, and *Lactobacillus acidophilus*. The powder can be used as a dietary fiber as well as a prebiotic [176]. Hydrothermal decomposition of heteropolysaccharides to oligosaccharides has also been shown to have a potential prebiotic effect on the strains *B. longum* subsp. *infantis*, *B. animalis* subsp. *lactis*, *L. acidophilus*, and *L. rhamnosus* [180]. Efforts have also been made to create a biodegradable film with *Opuntia* mucilage. Films made from mucilage, glycerol, and polyvinyl alcohol (PVA) and chitosan showed a homogeneous structure and higher hydrophilicity and water absorption than films made with PVA or chitosan [181]. In a study by Allegra et al., *Opuntia ficus*-*indica* was used as a coating on figs. The coated fruit had less weight loss, longer freshness retention, and lower *Enterobacteriaceae* counts over 10 days of storage compared with uncoated fruit [182]. With the increasing demand for natural alternatives to many artificial products, research is also being conducted on nanoencapsulation of natural dyes, which have positive properties for human health. However, many factors (temperature, pH, light, oxygen access) can affect their stability. A study by de Campo et al. demonstrated the potential use of *Opuntia monacantha* mucilage as a nanoencapsulation material. The mucilage showed resistance to temperature (25 °C and 40 °C) and protected against zeaxanthin degradation (higher retention after 28 days compared with the control sample) [183]. Microencapsulation of betalains from *Opuntia ficus-indica* fruit by spray drying cladode mucilage resulted in reduced moisture content, retention above 70%, and increased fiber content [184]. *Opuntia* mucilages are used in small farms to purify water. Cladodes are used to treat sewage [41]. In a study by Adjeroud et al., prickly pear mucilage was used to remove copper from water by the electrocoagulation-electroflotation method with 100% efficiency [185]. All these studies suggest the eco-friendly potential of using mucilage in functional foods, food, and pharmaceuticals, as well as in wastewater treatment.

### 4.11. Linum usitatissimum

One of the most well-researched mucilaginous plants is *Linum usitatissimum*, which belongs to the genus *Linum* in the family *Linaceae*. This plant is known as linseed (when grown for the oil) as well as flaxseed (when grown for fiber). Due to its high utility, flaxseed is very abundant around the world and is often used as a fiber source. Linseed is an important oilseed crop used in food, feed, and other industrial applications. Extensive research has explored the medical benefits of linseed, including anti-cancer [186], anti-inflammatory, and anti-ulcer effects. Linseed has been demonstrated to reduce glucose concentration in blood and lower cholesterol [187]. It is also an anti-atherogenic agent, fighting against cardiovascular and obesity disorders [188]. A peptide found in flaxseed, cyclolinopeptide A, has been proven to possess antimalarial activity and to be immunosuppressive [189].

Linseed, contains approximately 35–45% fat (mainly unsaturated α-linolenic acid), 20–25% protein, 28–30% fiber, 8% moisture, and 3–4% ash. Flaxseed mucilage (FM) is a soluble dietary fiber, which composes approximately 3.0–15.0% of the seed mass. Numerous studies have investigated the chemical composition of FM and various methods of extractions. It was found that FM has a heterogeneous structure mainly composed of polysaccharides (80%) and protein (9%). Flaxseed mucilage contains two fractions. The acidic fraction (~25%) is composed of galacturonic acid (21.0–36.0%), rhamnose (11.0–16.0%), galactose (12.0–16.0%), and fucose (up to 5%). The neutral fraction (~75%) is composed of xylose (19.0–38.0%), arabinose (8.0–13.0%), and glucose (4.0–6.0%) [190,191,192].

Flaxseed mucilage is characterized by high water-holding capacity, amphiphilic properties, and foamability, as well as by molecular weight- and temperature-dependent emulsification properties. Flaxseed mucilage has become a common vegan substitute for egg white (aquaflaxa or aqua-flaxa) in bakery products, and has been applied in the food industry as a food additive [190]. Freeze-dried FM has been applied as an effective structure agent in gluten free-bread [69]. Linseed mucilage has also been proposed as a fat-replacer prebiotic in cream cheese [193].

Bustamante et al. used FM blended with flaxseed-soluble protein for encapsulation of *Lactobacillus acidophilus* La-05 by spray drying [194]. The mixture increased LAB cell viability. Flaxseed mucilage has also been found to be useful in the production of mucoadhesive microspheres for oral drug delivery, improving drug bioavailability by avoiding the hepatic first-pass effect as well as masking the bitter taste of medicine [195]. Several studies have confirmed that FM can be used to formulate physically stable but elastic and biodegradable food-coating films when blended with glycerol (as a plasticizer) or polyvinyl alcohol [196,197].

### 4.12. Aloe vera

*Aloe barbadensis*, popularly known as *Aloe vera*, is a plant classified as a succulent belonging to the *Asphodelaceae* family, *Aloe* genus [64,198]. It is one of the most well-known genera, with over 360 species [199]. A drought-tolerant plant, *Aloe vera* grows in tropical and subtropical countries, in dry and hot areas. It originates in Africa, India, China, as well as the Mediterranean regions. It is also found in the Canary Islands, Sicily, Malta and Cyprus. *Aloe* plantations are present in Barbados and the USA [199,200]. *Aloe vera* has stems (60–100 cm in length) from which grow fleshy, thick, green leaves with thorns. The leaves consist of an outer layer (exocarp), a middle layer (pulp), and an inner layer (gel-like pulp) [200,201].

Due to its rich chemical composition of 75 compounds, *Aloe* has a wide range of applications, mainly in the cosmetic, pharmaceutical, and food industries, and in beverages, creams, lotions, shampoos, and conditioners, in the form of gels and capsules [64,200]. It is known for its traditional uses to treat burns, inflammation, wounds and gastrointestinal ailments [200]. Water constitutes 98.5% of the pulp and 99.5% of the mucilage. The rest of the chemical composition of the pulp and gel consists of polysaccharides (acetylated mannan, mannan, acetylated glucomannan, glucogalactomannan, galactan, galactogalacturan, arabinogalactan, pectin, cellulose, glucose, L-rhamnose), anthraquinones (aloin, Aloe-emodin, isobarbaloin anthranol, cinnamic acid ester, aloetic acid), vitamins (thiamine, riboflavin, pyridoxine, L-ascorbic acid, β-carotene, choline, folic acid, α- tocopherol), minerals (Ca, Cr, Cu, Fe, Mg, K, P, Na, Zn, Cl), amino acids (glycine, alanine, valine, leucine, isoleucine, proline, hydroxyproline, threonine, methionine, phenylalanine, tyrosine, aspartic acid, glutamic acid, lysine, arginine, histidine), enzymes (catalase, amylase, alkaline phosphatase, lipase, carboxypeptidase, oxidase, cyclooxygenase, superoxide dismutase), arachidonic acid, γ-linolenic acid, steroids, gibberellins, triglycerides, triterpenoid, lignins, salicylic acid, and lecithin [202,203]. The presence of aloin in the pulp causes a bitter taste and can have a laxative effect. To obtain the pure pulp, the leaves should be peeled, washed, and pressed. Methods of extraction include separation of the pulp from the leaf peel, filtration, centrifugation or gel squeezing, heating steps, and the use of organic solvents (ethanol, dimethyl sulfoxide) [204,205,206]. In order to minimize the decomposition of polysaccharides under high temperature, Liu et al. analyzed the optimal ultrasonic extraction conditions without reducing the activity of the compounds. The optimal conditions were found to be under ultrasound at 500 W, at 70 °C for 60 min [207].

Saini et al. demonstrated the antioxidant properties of *Aloe vera* gel. Mice were exposed to gamma irradiation and given gel doses of 250, 500, and 750 mg/kg body weight. Delayed and milder effects were observed, as well as neutralization of free radicals (nitric oxide—NO, 2,2-diphenyl-1-picrylhydrazyl—DPPH^•^, 2,2′-azinobis-(3-ethylbenzothiazoline-6-sulfonic acid)—ABTS^•+^) [208]. The anticancer and anti-inflammatory properties of *Aloe vera* mucilage were demonstrated by Im et al. [209]. An 80% reduction in adenoma was observed in mice with colon cancer when they were administered processed *Aloe vera* gel (400 mg/kg). Western blot analysis showed inhibition of nuclear factor kappa B activity and expression, as well as phosphorylation of the activator of transcription 3, which is correlated with cancer and inflammation [209]. The anticancer properties of *aloe* extract were tested by Hussain et al. [210]. After 24 h treatment of MCF-7 and HeLa cancer cell lines with *Aloe vera* extract, the IC_50_ was 60%.

*Aloe vera* gel has also been proven to have antimicrobial properties. *Aloe vera* ethanol extract was found to inhibit the growth of *Enterococcus bovis*, *Staphylococcus aureus*, *Proteus vulgaris*, *Proteus mirabilis*, and *Morganella morganii* [211], as well as *P. aeruginosa* strains isolated from patients with skin burns (MIC ≤ 400 μg/mL) [212]. Antimicrobial properties were also observed against the oral pathogens *Aggregatibacter actinomycetemcomitans*, *Clostridium bacilli*, *Streptococcus mutans*, and *S. aureus* (extract concentration 50 and 100%) using the disc-diffusion method [205]. According to Quezada et al., acemannan and fructans present in *Aloe vera* may have prebiotic effects due to their fermentation by the probiotic bacterial strains *Lactobacillus* spp. and *Bifidobacterium* spp. [213]. Positive results were obtained by Gullón et al. using donor intestinal microbiota for fermentation of *Aloe vera* [214]. According to a study by Passafiume et al., *Aloe vera* mucilage can be used as an edible food coating. *Aloe vera* gel with lemon essential oil and a variant with gelling agent had a positive effect on quality parameters (firmness, color, weight). These variants also reduced the multiplication of microbes level due to gas exchange capability of the natural coating as well as the antimicrobial properties of the essential oils and *Aloe vera* mucilage [215].

### 4.13. Solanum betaceum

*Solanum betaceum* (syn. *Cyphomada betacea*) is commonly known as tamarillo or tree tomato. Tamarillo is native to the Andes, especially Peru, Ecuador, and Colombia, but is also cultivated in Indonesia, the Philippines, Malaysia, Thailand, Vietnam, and Papua New Guinea [216,217]. There are three main types of tamarillo, which vary in terms of the color of the ripe fruit skin: yellow, red, and purple [218].

Tamarillo fruits are low in carbohydrates, but rich in vitamins (especially B6, C, and E) and minerals (K, Mg, P, Ca, Fe, Zn, and Cu). Tamarillo contains approximately 3% fiber and many bioactive substances, such as anthocyanins (i.e., pelargonidin 3-rutinoside, pelargonidin 3-glucosyl glucose, cyanidin 3-rutinoside, cyanidin 3-glucoside, delphinidin 3-rutinoside, delphinidin 3-glucoside, and pelargonidin 3-glucoside), carotenoids (α-carotene, β-carotene, and β-cryptoxanthin), and flavonoids [217,219,220,221]. Nascimento et al. compared the polysaccharides in pulp and mucilages from ripe tamarillo fruits. The tamarillo mucilages contained highly methoxylated homogalacturonans mixed with type I arabinogalactans, a linear (1 → 5)-linked α-L-arabinan, and a linear (1 → 4)-β-D-xylan that was present only in the mucilage fraction [49]. Gannasin et al. detected two types of hydrocolloids in tamarillo, with different physicochemical properties. The first type was a low-molecular-weight arabinogalactan protein-associated low methoxyl pectin that was located in the seed mucilage. The second type comprised high-molecular-weight hemicellulosic polysaccharides located in the pulp [222]. The structure of the seed mucilage hydrocolloid is more branched and hydrophilic than that of the pulp [216]. Because of their different properties, the possible applications of these two types of tamarillo hydrocolloid are also different.

Gannasin et al. investigated the health benefits tamarillo seed mucilage. In studies conducted in vitro, the seed mucilages were shown to demonstrate the prebiotic activity. The seed mucilages were resistant to digestive enzymes, stimulated the growth of beneficial intestinal microbiota (i.e., *Lactobacillus* spp. and *Bifidobacterium* spp.), and inhibited the growth of some pathogenic bacteria [218]. In further studies, the seed mucilage hydrocolloids were found to bind bile acids. The capturing of bile acids by hydrocolloids in the small intestine and their excretion in the feces is considered to be one of the main mechanisms by which the hydrocolloid lowers cholesterol [223]. However, the mechanism of binding bile acids mediated by seed mucilage hydrocolloid is not yet fully understood [216]. According to Gunness and Gidley, hydrocolloids may form a barrier preventing bile acids from reaching the intestinal cells, or catch bile acids due to gelatinous cross-linking [223]. The seed mucilage hydrocolloids also exhibit foaming capacity, comparable to commercially used preparations. Studies have shown that they can maintain the 80% foam volume for 2 h, which indicates the possibility of using tamarillo seed mucilages as foam stabilizers and food emulsifiers in foam-based food products such as mousses, meringues, marshmallows, and foamy beverages [216].

### 4.14. Cydonia oblonga

*Cydonia oblonga* belongs to the *Rosaceae* family. Its common name is quince. The trees are commonly cultivated in the Middle East and South Africa, as well as in Central Europe [224]. The plant is characterized by quite large, yellow fruits with an asymmetrical shape. Each fruit contains 10 oval and reddish-brown seeds [224,225]. Quince is rich in many bioactive substances including phenolic acids, flavonoids, and antioxidants. It has many pro-health benefits and is high in nutritional value. In folk medicine, quince fruits and seeds have been used in the treatment of gastrointestinal diseases [226]. However, its raw fruits are underutilized [224]. Recent research has revealed a wide range of possible applications for quince seed mucilage (QSM) as a natural hydrogel obtained after soaking in water [225]. The phenolic profile of quince seeds includes caffeoylquinic acids, lucenin-2, vicenin-2, stel-larin-2, isoschaftoside, schaftoside, 6-C-pentosyl-8-C-glucosyl chrysoeriol, and 6-C-glucosyl-8-C-pentosyl chrysoeriol. Six different organic acids have been detected in quince seeds: ascorbic acid, citric acid, fumaric acid, malic acid, quinic acid, and shikimic acid [227].

Quince seed mucilage is composed of cellulose and water-soluble polysaccharides, mainly partially *O*-acetylated (4-*O*-methyl-D-glucurono)-D-xylan [228]. It has a high molecular weight of 9.61 × 10^6^ g/mol, which is higher than commercially available gums such as xanthan gum, guar gum, and gellan gum [229]. Rezagholi et al. showed that QSM contains 85.04% carbohydrates, 13.16% uronic acid, 2.78% protein, 5.64% ash, 0.75% fat, and 5.77% moisture. D-xylose accounts for 40.43% of the carbohydrates, D-mannose 31.11%, arabinose 6.39%, D-glucose 5.75%, and D-galactose 5.60%. Xylan and/or mannan forms the backbone, while arabinose, glucose and galactose are branches in the mucilage structure [229].

Nikoofar et al. applied QSM as a fat replacer in yogurt obtained from homogenized milk (2.5%). Semi-fat yogurt containing QSM was compared with full-fat samples without mucilage. The addition of QSM had no significant effect on acidity, color, or texture properties (adhesiveness, cohesiveness, and springiness), but increased thickening and decreased syneresis [230].

Quince seed mucilage has been the subject of many studies exploring its potential use as a novel biodegradable edible coating for various food products. Farahmandfar et al. used QSM as a coating agent on dried banana slices. The application of the QSM-based film led to a reduction in shrinkage, a reduction in the browning index, and an increase in the rehydration process [231]. Kozlu and Elmaci investigated edible QSM coatings as innovative methods for protecting mandarin fruits against weight loss and softening, as well as against color changes during storage [73]. Jouki et al. studied the physical and mechanical properties of QSM-based films (0.5–1.5%) with different glycerol concentrations (25–50% *w/w*). The films were characterized by hydrophilic properties and provided a good barrier, making them suitable for food packaging [224,232]. Films based on QSM based films could extend the shelf-life of food and significantly improve its quality during storage. It has been shown that QSM film reduces color changes, texture, and lipid oxidation in rainbow trout fillets during 18 days storage at 4 °C [232,233]. Additionally, the QSM-based film inhibited growth of *Escherichia coli*, *Shewanella puterfaciens*, *Yersinia enterolitica*, and *Staphylococcus aureus*. The antibacterial activity of QSM coatings containing thyme essential oil against gram-positive (*Staphylococcus aureus*, *Lactiplantibacillus plantarum*, *Listeria monocytogenes*, and *Bacillus cereus*) and gram-negative (*Escherichia coli*, *Yersinia enterocolitica*, *Pseudomonas aeruginosa*, *Salmonella* Typhimurium, *Shewanella putrefaciens*, and *Vibrio cholerae*) bacteria was confirmed by Jouki et al. [234].

Quince seed mucilage has been reported to have important pharmaceutical, medicinal, and cosmetic applications. It is used to heal wounds and treat inflammatory skin conditions (e.g., atopic dermatitis) as well as protect against skin toxicity caused by the T-2 toxin [75].

## 5. Summary and Future Perspectives

This review summarized the chemical, biological, and technological data regarding plant mucilages, with special attention to their nutraceutical, functional, and medical applications. The nutraceutical and functional food market is one of the fastest-growing food segments worldwide, and there is renewed interest in plant-based compounds with pro-health benefits, including as alternatives to fat and gluten (*Salvia hispanica*, *Ocimum basilicum*, *Plantago ovata*, *Cydonia oblonga*).

In the last decade, plant-based mucilages have attracted increasing attention due to their desirable attributes. They can be used as texturizing and stabilizing agents (*Salvia hispanica*, *Ocimum basilicum*, *Hyptis suaveolens*, *Trigonella foenum-graecum*, *Cassia uniflora*, and *Opuntia ficus-indica*) in a wide range of food and beverages. In addition to its interesting rheological properties, plant mucilage is eco-friendly, biodegradable, and possesses good antioxidant activity. More research is needed to better understand the phytochemicals found in plant mucilage and its potential ethnopharmacological uses. Plant mucilage shows good potential not only as a functional food, but also for the treatment of different metabolism-related diseases, including polycystic ovarian syndrome and diabetes (*Ocimum basilicum*). Pre-clinical and clinical trials should be continued. More research is also needed on the anti-nutritional aspects of some mucilages (*Linum usitatissimum*).

Some plant-derived mucilages have natural antimicrobial activity, and can also serve as a matrix to introduce other antimicrobials for the production of food, cosmetics, health-care materials, and packaging in the form of edible films, but also as aerogels (*Aloe barbadensis*, *Cydonia oblonga*, *Trigonella foenum-graecum*, *Spinacia olerace*). It is important to explore the antimicrobial properties of a broad spectrum of plant mucilages and to identify the optimal extraction and storage conditions to ensure the highest biological activity. The potential use of mucilages as drug-delivery (*Aloe barbadensis*, *Cassia tora*, *Basella alba*) carriers should also be explored more thoroughly.

## Figures and Tables

**Table 1:** Sources of plant-derived mucilages.

Family	Species	Common Name	Part of Plant	Mucilage Chemical Composition (Key Polysaccharides)	References
*Lamiales* order					
*Lamiaceae*	*Salvia hispanica* * Salvia macrosiphon *	Chia	Seed	Tetrasaccharide consisting of β-D-xylopyranosyl, α-D-glucopyranosyl, and 4-*O*-methyl-α-D-glucopyranosyluronic acid	[4]
*Lamiaceae*	*Ocimum basilicum* *	Great basil	Seed	Two major fractions of glucomannan and (1 → 4)-linked xylan and a minor fraction of glucan	[5,6]
*Lamiaceae*	*Hyptis suaveolens* *	Pignut, chan, bushmint, sangura, wilaiti tuls	Seed	Neutral fraction: D-galactopyranosyl-, D-glucopyranosyl- and D-mannopyranosyl-units; the acid polysaccharide: L-fucopyranosyl, D-xylopyranosyl and 4-*O*-methyl-D-glucuronic acid units	[7,8]
*Plantaginaceae*	*Plantago psyllium* * Plantago major * Plantago ovata *	Psyllium, ispaghula	Seed husk	Xylan with 1 → 3 and 1 → 4 linkages containing arabinose and xylose on the sides; other units found: arabinose, rhamnose, galactose, glucose and small amounts of mannose, galactose, galacturonic and glucuronic acids; branched structure	[9,10]
*Scrophulariaceae*	*Verbascum* spp.	Mullein	Flower	Pectic polysaccharide containing the rhamnogalacturonan	[11,12]
*Fabales* order					
*Fabaceae*	*Glycyrrhiza glabra*	Licorice root	Root	Arabinogalactan with saponins (including glycyrrhizin), flavonoids, isoflavones, coumarins, lactones, sterols	[13,14]
*Fabaceae*	*Cyamopsis tetragonoloba*	Guar, cluster bean, gavar, guvar bean	Seed	Complex polymer of galactose and mannose (galactomannan)	[15]
*Fabaceae*	*Trigonella foenum-graecum* *	Fenugreek	Seed	Galactomannan	[16]
*Fabaceae*	*Cassia obtusifolia* * Cassia fistula *	Sicklepod	Seed	Glucomannan	[17,18,19]
*Brassicales* order					
*Moringaceae*	*Moringa oleifera*	Kelor horseradish tree, drumstick, sajna	Bark	Arabinogalactan	[20,21]
*Brassicaceae*	*Arabidopsis thaliana*	Thale cress, mouse-ear cress	Seed	Unbranched rhamnogalacturonan with small quantities of homogalacturonan, cellulose, and arabinoxylan	[22]
*Brassicaceae*	*Sinapis alba*	Yellow mustard	Seed	Pectic polysaccharides and a small portion of β-1,4 linked glucosyl backbone	[23]
*Brassicaceae*	*Eruca sativa*	Arugula, garden rocket, rocket salad, roka, roquette, rucola or rugula	Seed	Polysaccharides built with mannose and galactose as main components with minor amount of fructose, glucose and arabinose	[24,25]
*Malvales* order					
*Malvaceae*	*Althaea officinalis*	Marshmallow	Root	Neutral and acidic polysaccharides, rhamnogalacturonan	[26,27]
*Malvaceae*	*Corchorus olitorius*	Jew’s mallow, molokhiajute plant	Leaf	Rhamnogalacturonan	[28,29,30]
*Malvaceae*	*Hibiscus rosa-sinensis*	Chinese hibiscus, China rose, rosemallow, shoeblackplant	Leaf	Backbone chain composed of alpha-1,4-linked D-galactosyl α-1,2-linked L-rhamnosyl α-1,4-linked D-galacturonic acid units	[31,32]
*Malvaceae*	*Abelmoschus esculentus*	Okra, Lady’s finger, green ginseng	Fruit	Rhamnogalacturonan	[33,34]
*Caryophyllales* order					
*Basellaceae*	*Basella alba* *	Malabar spinach	Leaf, stem, flower	Polysaccharide, containing D-galactose (as major monosaccharide) and L-arabinose (in minor amounts)	[35]
*Amaranthaceae*	*Spinacia oleracea* *	Spinach	Leaf	Polysaccharide, containing neutral sugar (arabinose, galactose, mannose, glucose, rhamnose and xylose) and uronic acids	[36,37]
*Talinaceae*	*Talinum triangulare* syn. *T fructiosum* *	Surinum purslane, Ceylon spinach, waterleaf	Leaf	Polysaccharide-proteins and polysaccharide-triterpenoids	[38,39]
*Cactaceae*	*Opuntia* spp. *	Nopal	Leaflike steam	Backbone of α-D-galacturonic acid units linked 1 → 2 to β-L-rhamnose units linked 1 → 4 with branching on C-4	[40,41]
Other orders (*Boraginales*, *Ericales*, *Malpighiales*, *Asparagales*, *Solanales*, *Dioscoreales*, *Rosales*)					
*Boraginaceae*	*Cordia dichotoma*	Indian cherry, fragrant manjack, snotty gobbles, cummingcordia, glue berry, pink pearl, bird lime tree	Fruit	Polysaccharide built of galactose, arabinose and glucuronic acid with traces of rhamnose	[42,43]
*Ebenaceae*	*Diospyros melanoxylon*	Coromandel ebony East Indian ebony	Seed, bark	Neutral arabinoxylans and acidic pectin-like rhamnogalacturonan	[44,45]
*Ebenaceae*	*Diospyros peregrina*	Gaub persimmon, Malabar ebony, Wild mangosteen, Indian persimmon	Fruit	Neutral arabinoxylans and acidic pectin-like rhamnogalacturonan	[45]
*Linaceae*	*Linum usitatissimum* *	Flaxseed, linseed	Seed	Neutral arabinoxylans and acidic pectin-like rhamnogalacturonan	[46,47]
*Asphodelaceae*	*Aloe barbadensis* *	Aloe vera burn plant, medicinal aloe or Barbados aloe	Leaf	Backbone of α-D-galacturonic acid units linked 1 → 2 to β-L-rhamnose units linked 1 → 4 with branching on C-4	[48]
*Solanaceae*	*Solanum betaceum*, syn. *Cyphomandra betacea* *	Tamarillo	Fruit	Methoxylated homogalacturonans mixed with type I arabinogalactans, a linear (1 → 5)-linked α-L-arabinan, and a linear (1 → 4)-β-D-xylan	[49]
*Dioscoreaceae*	*Dioscorea polystachya*	Chinese yam, cinnamon-vine	Bulb	Poly(β1-4) mannose with additional linkages and proteins mixed composition of mannose, glucose, galactose and glucuronic acid	[50]
*Rosaceae*	*Cydonia oblonga* syn. *Cydonia vulgaris* *	Quince	Seed	Glucuronoxylan based polysaccharides	[51,52]

* Plant species marked with an asterisk are discussed in detail in this article.
